# Effects of Lighting Interventions to Improve Sleepiness in Night-Shift Workers: A Systematic Review and Meta-Analysis

**DOI:** 10.3390/healthcare10081390

**Published:** 2022-07-26

**Authors:** Chi-Jen Wu, Tai-Yang Huang, Su-Fei Ou, Jen-Taie Shiea, Bih-O Lee

**Affiliations:** 1 College of Nursing, Kaohsiung Medical University, Kaohsiung City 80708, Taiwan; iris189212@gmail.com; 2 Kaohsiung Armed Forces General Hospital Zuoying Branch, Kaohsiung City 81342, Taiwan; 3 Nursing Department, Chi Mei Hospital, Tainan City 71004, Taiwan; u109840007@gap.kmu.edu.tw; 4 Nursing Department, Kaohsiung Medical University Hospital, Kaohsiung City 80708, Taiwan; sufei985400015@gmail.com; 5 Department of Chemistry, National Sun Yat-Sen University, Kaohsiung City 80424, Taiwan; jetea@mail.nsysu.edu.tw

**Keywords:** night-shift worker, healthcare worker, light, sleepiness, meta-analysis, nurses

## Abstract

Shift work disrupts an otherwise normal circadian rhythm, which may result in sleepiness among night-shift workers. Artificial light has been shown to alter the light–dark cycle of shift workers and reset or phase shift the biological clock, improving nighttime alertness in workers. However, the effect of light therapy on improving sleepiness in nighttime workers has not been effectively confirmed in nursing clinical studies, and it is worth using relevant studies to provide the best evidence in any clinical setting. Systematic review and meta-analysis were used. The study was performed using PRISMA. Academic Search Complete, Embase, MEDLINE, the Cochrane Library, and CINAHL were searched, from the inception of each database to 27 December 2021. The Cochrane risk of bias tool was used to assess the methodological quality of each study. Standardized mean differences (SMDs) with 95% confidence intervals (CIs) were synthesized using a random-effects model to assess the efficacy of lighting intervention to improve sleepiness in night-shift workers. Sensitivity analysis followed by subgroup analysis was employed to examine heterogeneity. The meta-analysis was performed using Review Manager 5.4.1 software. A total of 14 studies from 7 countries were included. The overall result shows that lighting interventions significantly improved sleepiness. Further, the blue-enriched white light with a color temperature greater than 5000 Kelvin was effective in improving sleepiness of night-shift workers. This study unveils the emergent knowledge that light interventions with blue-enriched white were effective in improving sleepiness for night-shift workers, including nurses. This finding can be applied to ensure patient safety, reduce accidents, and improve work efficiency and job satisfaction. Nurses constitute the largest health professional workforce. We suggest that hospitals can insert blue-enriched white light equipment for night-shift healthcare providers. Several evidence-based suggestions are made for further consideration.

## 1. Introduction

Shift workers are estimated to account for more than 25%, 20%, and 19.7% of the workforce in the US, the EU, and Taiwan [1,2,3], respectively. Lighting is essential for around-the-clock shift work that allows people to meet their needs. More than 20% of shift workers experienced shift-work-related disorders, including sleep disorders [4]. Shift work sleep disorder is characterized by daytime insomnia, sleepiness during night work, cognitive impairment, poorer work efficiency, and decreased quality of life [5].

Shift work is often associated with higher pressure for sleep at night as well as melatonin secretion [6]. Melatonin is mainly synthesized during the evening, reaches peak concentrations at night, and gradually diminishes during the day [7]. For example, Vanttola et al. (2020) found that night-shift healthcare providers experienced sleep disorders, sleepiness, physical and mental fatigue, and depression, resulting in poorer memory and decreased cognitive performance [8]. Research has demonstrated the significant impacts of shift work on health, such as a higher risk of developing cancer, cardiovascular disease, gastrointestinal disorders, diabetes, and metabolic disorders [4,9]. In healthcare settings, night-shift healthcare professionals are at a higher risk for occupational safety and hazard concerns, provide a poorer quality of care, and have lower job satisfaction [10]. Therefore, the circadian rhythm disturbance caused by night shifts and the problem of sleepiness at night work are important issues that need to be addressed and solved.

Lighting interventions delay the circadian rhythm at 4 to 5 a.m., when the body’s core temperature is at its lowest, whereas later exposure to light advances the circadian rhythm [11]. The retinal ganglion cells (RGCs) consist of cone and rod cells as well as intrinsically photosensitive retinal ganglion cells (ipRGCs), which account for only 2% of all RGCs. These melanopsin-rich ipRGCs are intrinsically photosensitive and have non-visual and photoreceptive properties. ipRGCs, which are highly sensitive to short-wavelength and high-temperature blue-enriched white light, can suppress melatonin secretion [12].

Previous studies have demonstrated that light was the most important and effective intrinsic factor affecting circadian rhythm and regulating the circadian rhythms of shift workers [13]. For example, Aemmi et al. (2020) published the first systematic review on the effects of light in improving nurse night-shift workers’ sleepiness. Due to the high heterogeneity of the five studies, the findings did not support the effects on improved sleepiness [14]. Lam and Chun (2021) conducted a systematic review and meta-analysis of 21 studies on the effects of light in reducing sleepiness among shift workers. The findings suggested that the optimal combination is a single, medium-dose (2000–5000 lux) session that lasts for less than one hour [15]. In addition to dose and duration, the light spectral distribution must be considered in light therapy [16]. Light, especially blue light, is said to indirectly affect an individual’s alertness through circadian rhythm changes and directly cause acute alerting effects [17]. This study aimed to assess the effects of light interventions in improving sleepiness and to analyze the effects of spectral characteristics on the improvement of sleepiness among healthcare and non-healthcare night-shift workers.

## 2. Methods

This study was conducted according to the Preferred Reporting Items for Systematic Review and Meta-Analysis (PRISMA) guidelines [18]. It has been registered on the PROSPERO online system (ID CRD42022339308).

### 2.1. Search Methods

A systematic search was performed to identify studies using the databases Embase, MEDLINE (Ovid), Cochrane Library, and CINAHL Plus with Full Text (EBSCOhost). The selected studies were randomized controlled trials or randomized crossover studies from database inception to 27 December 2021. The controlled vocabulary (MeSH) and text words combined main concepts (shift worker, light, photography, sleepiness, somnolence, and cognition) and keywords using Boolean operators: (shift worker OR shift work OR in shift OR night-shift OR irregular work OR rotating shift work OR a rotation system OR a relay system OR attendance OR work OR laborer OR labourer OR job) AND (photograph OR light OR photoinduction OR photon radiation OR photoradiation OR optical OR bright) AND (sleepiness OR drowsiness OR somnolence OR sleep disorder OR drowsiness OR particularly in excess OR vigilance OR alertness OR weather eye OR look-out OR cognitive; Appendix Table A1).

### 2.2. Search Outcome

The criteria for inclusion were based on population, intervention or issue of interest, comparison, outcome, and study design (PICOS) criteria [19]. The inclusion criteria were: (a) the participants had to be shift workers older than 18 years, (b) the intervention was light therapy, (c) the outcomes were the participants’ subjective and scale-assessed sleepiness, and (d) the study design included randomized controlled trials (RCTs) or crossover randomized studies. The exclusion criteria were non-English-language articles that did not satisfy the PICOS criteria or for which the full-text article was not available and posters.

### 2.3. Quality Appraisal

Two reviewers separately reviewed and extracted raw data from the 14 studies and verified them one by one. In the event of any inconsistency, a third reviewer was consulted to address the difference in opinion.

### 2.4. Data Abstraction

The characteristics and outcomes of a study were extracted based on the publication year, number of participants, country of origin, details of the intervention, and details of outcomes for the control group. The raw data (mean and standard deviation) for each study’s findings were recorded in an Excel spreadsheet (Microsoft, Redmond, WA, USA). The quality of each study included was then appraised by the two reviewers using the Cochrane Collaboration risk of bias tool (Cochrane, Oxford, UK) [20]. Following the assessment, the two reviewers reached a consensus on every item; if they did not agree, the third reviewer assessed the item and discussed it with the other two reviewers to reach a consensus.

### 2.5. Synthesis

In the meta-analysis, data were extracted using Cochrane Review Manager Version 5.4.1 software (Cochrane Centre, Oxford, UK) [21]. In one study [22], the maximum score of the Karolinska Sleepiness Scale (KSS) was set to 10 points, and the data were standardized to 9 points. Six studies presented their sleepiness scales as line graphs or bar charts whose data were extracted using the WebPlot Digitizer (WebPlotDigitizer, Pacifica, CA, USA) (Copyright 2010–2021 Ankit Rohatgi) tool, and Excel functions were used to generate the means and standard deviations. Considering that the studies analyzed used different sleepiness scales, Hedges’ (1981) concept was adopted to estimate the effect size of a single study by presenting the findings of various studies as the standardized mean difference (SMD) with 95% confidence interval (CI) [23]. The I^2^ statistic was used to test the heterogeneity between studies, with 0%, 25%, 50%, and ≥75% indicating no, low, moderate, and high heterogeneity, respectively [24]. The random-effect model analytical approach was used when the I^2^ exceeded 50% so that the variance between therapies was not underestimated. High heterogeneity was addressed through sensitivity analysis and subgroup analysis. A *p*-value of less than 0.1 indicated that the subgroup analysis was statistically significant [25], and a *p*-value less than 0.05 indicated that all analyses were statistically significant.

## 3. Results

### 3.1. Selection of Studies

**Figure 1:**
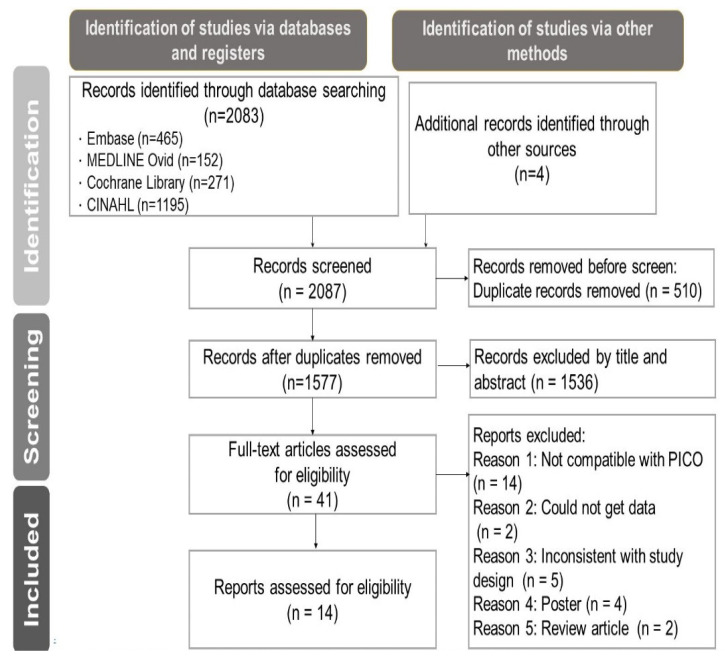
PRISMA flow diagram of study selection.

A preliminary search of the databases yielded a total of 2083 records, of which 510 were duplicates and removed by EndNote X9 (Clarivate Analytics, Chandler, AZ, USA). The titles and abstracts of the remaining 1573 studies were screened, and 1536 were removed. Of the remaining 37 studies, 27 were removed because the inclusion criteria were not met. The remaining 10 studies plus 4 that were manually retrieved from other sources were subjected to a quantitative systematic literature review and meta-analysis (Figure 1).

### 3.2. Description of the Included Studies

A total of 621 participants were included in 14 studies across 5 healthcare and 9 non-healthcare studies. Of the 14 studies, 4 were RCTs, while 10 were case-crossover studies. The lighting interventions in the experimental groups differed in lighting equipment, illuminance (in units of lux), lighting time points, exposure duration, spectrum wavelength, and color. Eight studies [22,26,27,28,29,30,31,32] applied lighting with a color temperature greater than or equal to 5000 K and three applied lighting with a color temperature below 5000 K [33,34,35]. In terms of sleepiness assessment, 12 studies adopted the KSS [11,13,22,26,27,28,29,30,31,32,34,36], and 2 [33,37] adopted the Stanford Sleepiness Scale (SSS), as seen in Table 1.

**Figure 2:**
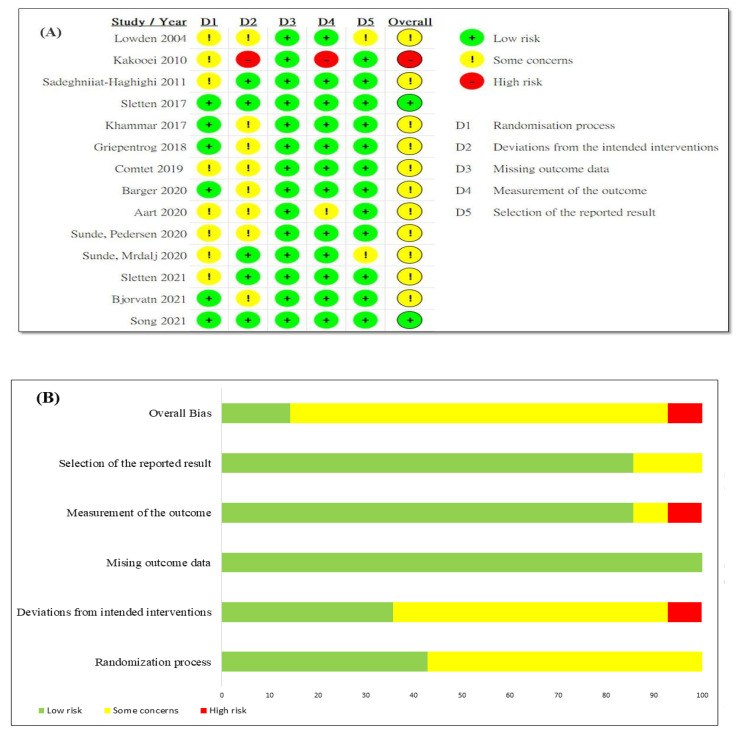
(**A**) Risk of bias summary [11,13,22,26,27,28,29,30,31,32,33,34,36,37]. (**B**) Risk of bias graph.

The risk of bias for each study, as shown in the randomization process and deviations from the intended interventions, shows some concerns for 57.1% (8/14), low risk of missing outcome data for 100%, and low risk in measurement of the outcome and selection of the reported result for 85.7% (12/14). The quality results show low risk for around 14.3%, some concerns for around 78.6%, and high risk for around 7.1%. The included 14 studies were classified as medium quality (Figure 2).

### 3.3. Risk of Bias and Sensitivity Analysis

**Figure 3:**
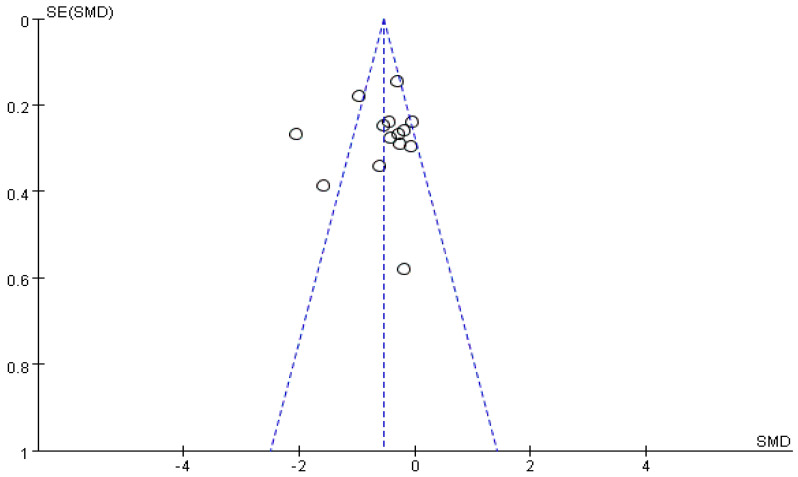
Funnel plot showing the results for sleepiness.

To guarantee the validity of the meta-analysis, risk of bias analysis was performed for each study, and the results are presented as a funnel plot with a fail-safe number (FSN). An asymmetrical funnel plot or an FSN lower than the tolerance level (TL) indicates the possibility of publication bias [38]. The funnel plot in this study is asymmetrical, because the effect sizes of the two studies [33,36] were beyond the two standard deviations of the combined average effect size (Figure 3). Sensitivity analysis was performed to remove the two studies with the highest effects (−0.39; 95% CI: −0.57, −0.22; χ^2^ = 16.06, *p* = 0.014, I^2^ = 32%), which resulted in low to moderate heterogeneity. The FSN test results show that the FSN of 96 was greater than the TL of 80, and no significant risk of bias was detected. The results are unaffected by any significant risk of bias.

### 3.4. Effects of Lighting Interventions in Improving Sleepiness

**Figure 4:**
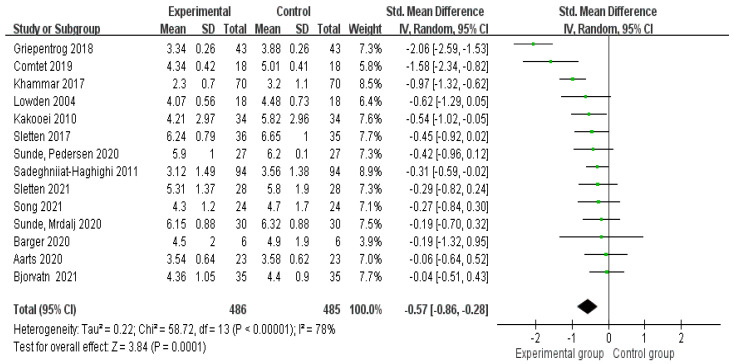
Forest plot of the effect of lighting interventions in improving sleepiness of night−shift workers [11,13,22,26,27,28,29,30,31,32,33,34,36,37].

Of the 14 studies included in this review, 5 [22,27,33,36,37] found that lighting interventions significantly improved sleepiness, while 9 [11,13,16,26,29,30,31,32,34] indicated that lighting interventions had no significant effects in improving sleepiness. High heterogeneity was reflected by the I^2^ of 78%. Thus, a random-effect model was used, with an SMD of −0.57 (95% CI: −0.86 to −0.28). In general, the result of the analysis show that lighting interventions significantly improved sleepiness. The total effect of the Z-test was statistically significant (Z = 3.84, *p* < 0.0001). Figure 4 shows the overall effect of lighting interventions in improving sleepiness.

### 3.5. Subgroup Analyses

According to the characteristics of the studies and the purpose of the research, the studies were divided into subgroup analyses to further understand the effect of light. The subgroup results are shown in Table 2.

- Effects of lighting interventions on different groups in improving sleepiness

**Figure 5:**
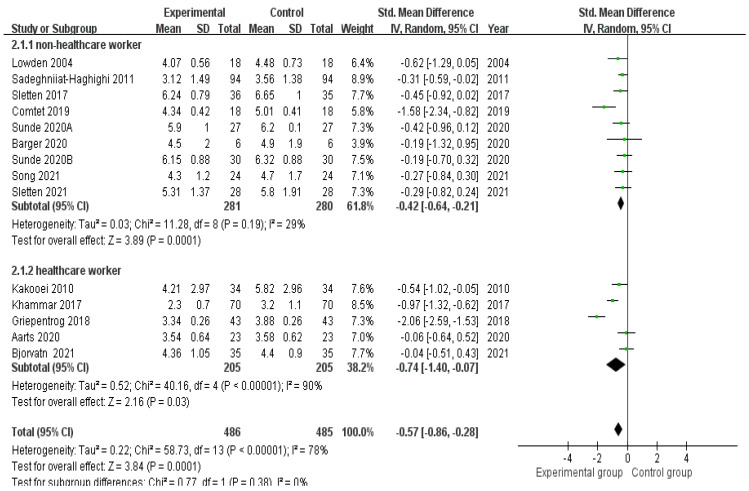
Effects of lighting interventions in improving the sleepiness of night-shift non-healthcare and healthcare workers [11,13,22,26,27,28,29,30,31,32,33,34,36,37].

Nine studies [16,26,29,30,31,32,34,36,37] showed that lighting interventions significantly (Z = 3.89, *p* = 0.0001) reduced the nighttime sleepiness of night-shift non-healthcare workers, with an SMD of −0.42 (95% CI: −0.64 to −0.21) and statistical heterogeneity being present (χ^2^ = 11.28, *p* = 0.0001, Tau^2^ = 0.03, I^2^ = 29%). Five studies [11,13,22,27,33] also showed that lighting interventions significantly (Z = 2.16, *p* = 0.03) reduced the nighttime sleepiness of night-shift healthcare workers, with an SMD of −0.74 (95% CI: −1.40 to −0.07) and statistical heterogeneity being present (χ^2^ = 40.16, *p* = 0.00001, Tau^2^ = 0.52, I^2^ = 90%; Figure 5).

- Effects of color temperature in different spectral characteristics

**Figure 6:**
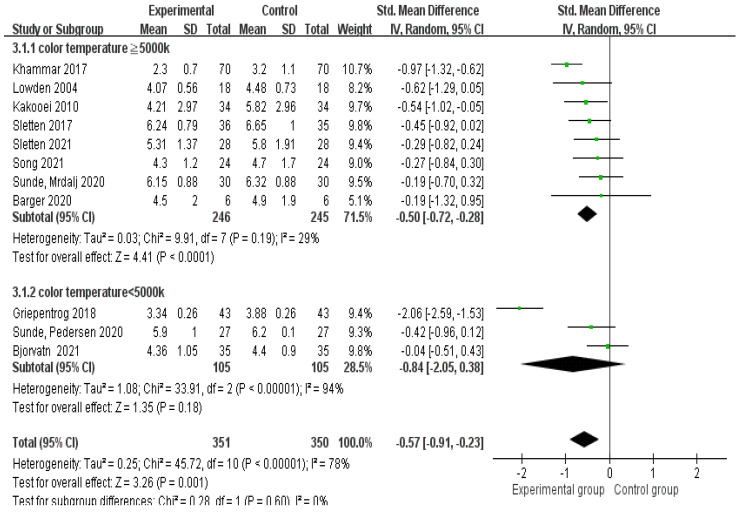
Compared effects of color temperature at least 5000 K and below 5000 K in improving the sleepiness of night-shift workers [11,22,26,27,28,29,30,31,32,33,34].

Eight studies [16,22,26,27,29,30,31,32] showed that lighting with a color temperature greater than or equal to 5000 K significantly (Z = 4.41, *p* = 0.0001) improved the sleepiness of night-shift workers, with an SMD of −0.50 (95% CI: −0.72 to −0.28) and statistical heterogeneity being present (χ^2^ = 9.91, *p* = 0.0003, Tau^2^ = 0.03, I^2^ = 29%). Three studies [11,33,34] showed that lighting with a temperature below 5000 K had no significant effect (Z = 1.35, *p* = 0.18) on sleepiness, with an SMD −0.84 (95% CI: −2.05 to 0.38) and statistical heterogeneity being present (χ^2^ = 33.91, *p* = 0.00001, Tau^2^ = 1.08, I^2^ = 94%; Figure 6).

## 4. Discussion

The emergent knowledge from this study is that blue-enriched white light with a color temperature of 5000 K or higher was effective in improving the sleepiness of night-shift workers. Furthermore, this study found that lighting interventions significantly improved the sleepiness of night-shift workers in healthcare and non-healthcare settings.

This study has confirmed that lighting interventions significantly improved sleepiness for night-shift workers. In reference to the previous inconclusive findings, this result confirms the stated purpose of this study. Thus, we recommend installing adequate lighting in night-shift settings such as nurse stations and employee rest areas to improve sleepiness and work performance of night-shift workers.

The finding from this study suggests that high-temperature lighting effectively improved sleepiness. This result differs from Lam and Chung’s study (2021), which examined dose-response effects on sleepiness and circadian rhythm changes of shift workers. The results recommended a dose range of 2000 to 5000 lux for effectively improving sleepiness among night-shift workers [15]. The reason for the difference in results may be that spectral attributes were not considered in the comparative study. Rodenbeck et al. (2018) found that different light intensities, colors, and wavelengths affected the mood and alertness of night-shift workers [39]. The spectral length and color of short-wavelength blue-enriched white light effectively suppressed melatonin secretion by the pineal body and improved subjective alertness and work performance [29]. Additionally, Motamedzadeh et al. (2017) found that compared with white light (4000 K), blue-enriched white light (17,000 K) had stronger effects on improving work performance, mood, alertness, and sleep quality [40]. Sletten et al. (2017) found that the application of low-intensity blue-enriched white light from the hours of 11 pm to 7 am improved the sleepiness of night-shift workers [29]. Importantly, this study demonstrated that blue-enriched white light with a temperature greater than 5000 K was significantly more effective than a temperature lower than 5000 K for improving sleepiness.

This study found that lighting significantly reduced the sleepiness of night-shift non-healthcare workers. Seven percent of workers reported sleeping on the job frequently every month, which resulted in work errors, abnormalities, and safety incidents [41]. The occurrence of prolonged sleepiness can lead to chronic health problems [16]. High-illuminance lighting has been demonstrated to reduce nighttime fatigue and sleepiness and improve the work performance of night-shift workers [42]. In addition, due to the different attributes of the electromagnetic spectrum, increasing the intensity and illuminance of blue-enriched white light stimulates melanopsin-dependent responses, rapidly and effectively improves the sleepiness of night-shift workers, and enhances their subjective alertness at work [30]. Based on these findings, a precise light intervention method may be inexpensive and potentially beneficial for healthcare providers such as nurses. Also, the socioeconomic status among variables or potential determinant of sleep health during lightning interventions may be considered for future studies.

The high heterogeneity of the results of this current study may be due to the differences in the designs and interventions used, such as participants (healthcare providers and laboratory-simulated night-shift workers), lighting equipment, duration of continuous lighting, time of lighting, location of lighting, length (in days) of lighting, and time of sleepiness evaluation. In addition, two studies with effect sizes that fell outside the standard deviation range of the combined average effect were further investigated. Thus, large-scale RTCs can be used to test either in clinical settings or industries to accrue evidence from different industries. Future research can also mediate shift schedules and control environmental factors that are crucial to the self-perceived competence of sleep-deprived shift workers in an RTC design.

A previous study adopted a randomized crossover trial design to examine night-shift nurses in a surgical intensive care unit (ICU) in the US [33]. The participants were exposed to light-emitting diode (LED) light and white light at an illuminance of 1500–2000 lux for 10 h from 1900 to 0500 h and were then evaluated for their sleepiness using a scale at 0500 h. As the approach differed from those of other studies that measured participants’ sleepiness at multiple time points, it may be the reason for the findings being inconsistent. In Comtet et al.’s study, participants wore 2000 lux blue-enriched white light optical glasses for 30 min at 0500 h, and their sleepiness was measured at 0500, 0700, and 0800 h [36]. The results differed significantly from others because of the study’s unique lighting intervention, time points of sleepiness measurement, and research design. Future research can re-examine the effects of blue-enriched white light with a temperature greater than or equal to 5000 K plus low to moderate light intensities for improving sleepiness among night-shift workers in clinical settings or industries.

## 5. Limitations

In this study, the experimental studies were analyzed together, which resulted in heterogeneity between the studies and potential over-representation of specific groups. While the quality of the studies reviewed was moderate, the unclear risk of bias was high. Due to the nature of the interventions, a higher level of bias was caused by randomization processes and deviations from intended interventions. Rigorous and large-scale designs should be adopted in future studies to address these biases. Moreover, this study used data from healthcare and non-healthcare studies. The applicability of the results in different industries may be limited. Given that nurses constitute the largest health professional workforce, it would be prudent to conduct large-scale RTCs to provide stronger evidence than is available from this study to retest the effects of lightning interventions on nurses.

## 6. Conclusions

This study indicates that lighting interventions can improve the sleepiness and alertness of night-shift workers. To ensure patient safety and reduce abnormal accidents, healthcare providers and non-healthcare work settings are recommended to employ lighting interventions involving blue-enriched white light and temperatures greater than or equal to 5000 K, effective in improving the sleepiness of night-shift workers. The results of this study can serve as a reference for healthcare institutions and other sectors to effectively deal with the problem of sleepiness of night-shift workers as well as increase productivity and job satisfaction.

## 7. Implications

The lighting interventions use non-pharmaceutical, ubiquitous, and economical equipment to mediate workers’ circadian rhythms. The improvement of sleepiness should positively affect patient safety and reduce industrial incidents. Several recommendations are made.

First, hospitals can use light interventions for night-shift healthcare providers using suggested illuminance rating and ipRGCs that are highly sensitive to short-wavelength and high-temperature blue-enriched white light. To quickly reduce drowsiness and improve alertness, the suggestion mentioned should be combined with color temperatures of at least 5000 K to directly suppress melatonin. A phototherapy device can be combined with spectral properties in clinical settings. Second, we suggest that hospital managers insert blue-enriched white light equipment in lounges, portable devices, computers, and work vehicles to increase nurses’ awareness of the relationships between sleepiness and light. For non-healthcare workers, similar strategies can be applied in different industries based on their availabilities and needs. Third, the lighting environments of night-shift workers also affect the effectiveness of lighting interventions. Quantitative assessment of environmental lighting before intervention implementation can provide a better basis for evaluating the effectiveness of lighting interventions. Last, on-the-job education can include the findings from this study in healthcare and non-healthcare settings to help raise night-shift workers’ awareness of their health.

## Figures and Tables

**Table 1:** Characteristics and outcomes of included studies.

Author/ Year/Country	Study Design	Intervention Group (IG)	Control/Contrast Group (CG)	Population	Duration	Measurement Time Point	Outcomes and Measurement Tools
Lowden (2004) Sweden [28]	Crossover design	E: 2500 lux (5000 K) The workers were permitted two short breaks at night in the break room and light exposure time from nighttime 24:00–6:00 at any time the workers chose in the break room. In total, 67% of the workers chose between 3:00 and 4:00. Average exposure time length 20 ± 0.48 min.	300 lux (3000 K)	Workers from a truck production plant 18	4 weeks	24:00–6:00 q2h*2 check on every W1~W4 night	A significant interaction. Demonstrated a reduction in sleepiness in the BL condition, particularly on the first two nights at 4:00 and 6:00 h. KSS
Sadeghniiat-Haghighi (2011) Iran [37]	Crossover design	E: 2500 lux The light exposure occurred during two short breaks during the night shift. Each break was approximately 20 min long. The break time started at 24:30 and 2:30.	300 lux	Workers from a ceramics factory 94	2 nights	22:00, 24:00, 2:00, 4:00 check	Exposure to bright light might be effective in reducing the sleepiness of night workers. SSS
Sletten (2017) Australia [29]	RCT	E: 89 lux (17,000 K) Laboratory procedures for two consecutive night shifts at their usual occupation; workers remained in the laboratory from 17:30 to 8:30 for a simulated night shift.	84 lux (4000 K)	Night-shift workers 71	2 nights	23:00–7:00 q1h check	There were no differences between light conditions based on time into a shift, but blue-enriched light improved subjective sleepiness when light exposure coincided with the trough of the circadian rhythm. KSS
Comtet (2019) France [36]	Crossover design RCT	E: 2000 lux Health workers were given LED blue-enriched white light glasses at 5:00 for 30 min.	Dim light 8 lux	Health workers 18	3 nights	5:00, 7:00, 8:00 check	Early morning light therapy under the condition of sleep loss may have broad practical applications to reduce sleepiness. KSS
Sunde, Pedersen (2020) Norway [34]	Crossover design	E: 900 lux (4000 K) The college students were given 3 nights of light exposure from 23:00 to 5:00.	90 lux (4100 K)	College students simulating shift workers 27	3 nights	23:30, 1:00, 2:30, 4:00,5:30 check	Bright light improved alertness. KSS
Barger (2020) USA [26]	RCT	E: 63 lux (8000 K) Subjects were given two 20 min breaks (11:00–3:30 and 3:30–8:00) and light exposure every night.	63 lux (4100 K)	Flight controllers 12	5 nights	11:00, 11:00–3:30, 3:30–8:00, 8:00 check	Short-wavelength light exposure was successful in improving alertness and performance. KSS
Sunde, Mrdalj (2020) Norway [31]	Crossover design RCT	E: 200 lux (7000 K) The college students were given exposure to polychromatic, blue-enriched white light from 23:00 to 5:00.	200 lux (2500 K)	College students simulating shift workers 30	3 nights	23:30, 1:00, 2:30, 4:00,5:30 check	7000 K light was more beneficial compared to 2500 K for subjective alertness during night shifts. KSS
Song (2021) China [32]	RCT	E: 320 lux (6000 K) Subjects were exposed to blue light from 22:00–7:00.	320 lux (3000 K)	College students simulating shift workers 48	1 night	22:00–24:00, 2:00–4:00, 6:00–7:00 check	Exposure to blue-enriched white light could reduce sleepiness. KSS
Sletten (2021) Australia [30]	RCT	E: 106 lux (17,000 K) Laboratory procedures for two consecutive night shifts; workers remained at the laboratory from 17:30 h until 08:30 h for a simulated night shift at their usual occupation.	43 lux (4000 K)	Night-shift workers 28	2 nights	23:00–7:00 q1h check	Blue-enriched light improved subjective sleepiness when light exposure coincided with the trough of the circadian rhythm. KSS
Kakooei (2010) Iran [22]	Crossover design	E: 4500 lux (5000 K) Nurses were exposed to light intervention during two breaks (21:15–22:00 and 3:14–4:00).	300 lux	Nurses from a hospital 34	3 nights	19:00–16:00 q1h check	BL intervention improved alertness. KSS
Khammar (2017) Iran [27]	Crossover design RCT	E: 3500 lux (5000 K) The workers were exposed to full-spectrum light tubes at four breaks during the night shift (24:00, 2:00, 4:00, 6:00) in the break room.	Dim 400 lux	Hospital Workers 140	2 nights	23:00–5:00 q2h check	Exposure to BL reduced shift workers’ sleepiness. KSS
Griepentrog (2018) USA [33]	Crossover design	E: 1500–2000 lux (3500–4100 K) Nurses were exposed to light intervention exposure from 19:00 to 5:00.	300 lux	Nurses from a hospital 43	1 night	5:00 check	BL intervention significantly reduced nurses’ sleepiness. SSS
Aarts (2020) The Netherlands [13]	Crossover design RCT	E: wear glasses FN (λ = 462 ± 10 nm, spectral irradiance of 22.36 µW/cm^2^/melanotic EDI of 114.38 lx at the cornea of the eyes) 4 × 15 min during nightshifts and for 30 min within 2 h after awakening.	LED 84 lux	Nurses from a hospital 23	3 nights	24:00, 2:00, 4:00, 6:00, 8:00 q2h check	Wearing glasses as light intervention significantly reduced nurses’ sleepiness on the first night shift. KSS
Bjorvatn (2021) Norway [11]	Crossover design	E: BL 10,000 lux (4000 K) Nurses were exposed to light for 30 min at 2:00–3:00, 3:00–4:00, and 4:00–5:00.	Red dim light 100 lux	Nurses from a hospital 35	3 nights	22:00–6:00 q2h check	No significant intervention effect was found for sleepiness. KSS

Note: BL, bright light; KSS, Karolinska Sleepiness Scale; EDI, electronic data interchange; LED, light-emitting diode; RCT, randomized controlled trial; SSS, Stanford Sleepiness Scale quality assessment of the included studies.

**Table 2:** Subgroup analysis of the efficacy of light in reducing sleepiness.

Subgroup	Effect Size				Heterogeneity		
	Number of Studies	SMD	(95% CI)	*p*	χ^2^	*p*	I^2^ (%)
Characterization of group							
Non-healthcare workers	9	−0.42	−0.64, −0.21	<0.0001	11.28	=0.019	29
Healthcare workers	5	−0.74	−0.14, −0.07	<0.03	40.16	<0.00001	90
Total between					58.73	<0.00001	78
Color temperature of light							
≥5000 K	8	−0.50	−0.72, −0.28	=0.0003	9.91	=0.13	29
<5000 K	3	−0.84	−2.05, −0.38	=0.18	33.91	<0.00001	94
Total between					45.72	<0.00001	78

Note: CI, confidence interval; I^2^, heterogeneity; SMD, standardized mean difference.

## Data Availability

Data will be extracted from papers.

## Appendix A

**Table A1:** Literature search strategy.

Database		Search Strategy
Embase	1.	(((“shift worker” OR “shift*work*” OR “in shift*” OR “Night Shift*” OR “irregular work*” OR “Rotating Shift Work” OR “a rotation system” OR “a relay system”) Near/4 (attendance OR work* OR laborer OR labourer OR job))):ti,ab,kw,de
	2.	“Shift worker”/exp
	3.	(photograph* OR light* OR photoinduction* OR “photon radiation*” OR photoradiation*; OR optical* OR bright*):ti,ab,kw,de
	4.	“light”/exp OR “photography”/exp
	5.	(Sleepiness* OR drowsiness* OR somnolence* OR “Sleep disorder*” OR drows* OR “particularly in excess” OR vigilance* OR alertness OR “weather eye*” OR “look-out*” OR cognitive*):ti,ab,kw,de
	6.	“Somnolence”/exp OR “Cognition”/exp
	7.	(#1 OR #2) AND (#3 OR #4) AND (#5 OR #6) [embase]/lim
MEDLINE (Ovid)	1.	((“shift worker” OR “shift*work*” OR “in shift*” OR “Night Shift*” OR “irregular work*” OR “Rotating Shift Work” OR “a rotation system” OR “a relay system”) Adj4 (attendance OR work* OR laborer OR labourer OR job)).mp
	2.	exp “Shift Work Schedule”/
	3.	(photograph* OR light* OR photoinduction* OR “photon radiation*” OR photoradiation* OR optical* OR bright*).mp
	4.	exp “Light”/OR “light exposure”/OR “phototherapy”/
	5.	(Sleepiness* OR drowsiness* OR somnolence* OR Sleep disorder* OR drows* OR “particularly in excess” OR vigilance* OR alertness* OR “weather eye*” OR “look-out*” OR cognitive*).mp
	6.	exp “Somnolence”/OR “Sleepiness”/OR “Cognition”/
	7.	(1 OR 2) AND (3 OR 4) *AND (5 OR 6)*
CENTRAL	1.	(((“shift worker” OR “shift*work*” OR “in shift*” OR “Night Shift*” OR “irregular work*” OR “Rotating Shift Work” OR “a rotation system” OR “a relay system”) Near/3 (attendance OR work* OR laborer OR labourer OR job))):ti,ab,kw
	2.	[mh “Shift Work Schedule”]
	3.	(photograph* OR light* OR photoinduction* OR “photon radiation*” OR photoradiation* OR optical* OR bright*):ti,ab,kw
	4.	[mh “Light”] OR [mh “phototherapy”]
	5.	(Sleepiness* OR drowsiness* OR somnolence* OR “Sleep disorder*” OR drows* OR “particularly in excess” OR vigilance* OR alertness OR “weather eye*” OR “look-out*” OR cognitive*):ti,ab,kw
	6.	[mh “Somnolence”] OR [mh “Sleepiness”] OR [mh “Cognition”]
	7.	(#1 OR #2) AND (#3 OR #4 OR #5 OR #6)
CINAHL	1.	(((“shift worker” OR “shift*work*” OR “in shift*” OR “Night Shift*” OR “irregular work*” OR “Rotating Shift Work” OR “a rotation system” OR “a relay system”) N3 (attendance OR work* OR laborer OR labourer OR job)))
	2.	MH (“Shift Work Schedule+”)
	3.	(photograph* OR light* OR photoinduction* OR “photon radiation*” OR photoradiation* OR optical* OR bright*)
	4.	MH (“Light+”) OR MH(“Phototherapy+”)
	5.	(Sleepiness* OR drowsiness* OR somnolence* OR Sleep disorder* OR drows* OR “particularly in excess” OR vigilance* OR alertness* OR “weather eye*” OR “look-out*” OR cognitive*)
	6.	MH (“Somnolence+”) OR MH (“Sleepiness+”) OR MH (“Cognition+”)
	7.	(S1 OR S2) AND (S3 OR S4 OR S5 OR S6)
Google Scholar	1.	((shift worker OR night shift OR irregular work OR Rotating Shift Work OR a rotation system OR a relay system) AND (photograph OR light OR photoinduction OR photon radiation OR optical OR bright) AND (Sleepiness OR drowsiness OR somnolence OR vigilance OR alertness OR weather eye OR look-out OR cognitive))
